# Probing of local polarity in poly(methyl methacrylate) with the charge transfer transition in Nile red

**DOI:** 10.3762/bjoc.15.248

**Published:** 2019-10-25

**Authors:** Aydan Yadigarli, Qimeng Song, Sergey I Druzhinin, Holger Schönherr

**Affiliations:** 1 Physical Chemistry I and Research Center of Micro and Nanochemistry and Engineering (Cμ), Department of Chemistry and Biology, University of Siegen, Adolf-Reichwein-Str. 2, 57076, Siegen, Germany

**Keywords:** charge transfer, dipole moment, fluorescence, inhomogeneous broadening, oxazine dye, polarity probe, polymer permittivity

## Abstract

The permittivity of polymers and its spatial distribution play a crucial role in the behavior of thin films, such as those used, e.g., as sensor coatings. In an attempt to develop a conclusive approach to determine these quantities, the polarity of the model polymer poly(methyl methacrylate) (PMMA) in 600 nm thin films on a glass support was probed by the energy of the charge transfer transition in the oxazine dye Nile red (NR) at 25 °C. The absorption and fluorescence spectra of NR were observed to shift to the red with increasing solvent polarity, because of the intramolecular charge transfer character of the optical transition. New types of solvatochromic plots of emission frequency against absorption frequency and vice versa afforded the Onsager radius-free estimation of the ground and excited states dipole moment ratio. With this approach the values of these dipole moments of 11.97 D and 18.30–19.16 D, respectively, were obtained for NR. An effective local dielectric constant of 5.9–8.3 for PMMA thin films was calculated from the solvatochromic plot and the fluorescence maximum of NR observed in the PMMA films. The fluorescence band of NR in the rigid PMMA films shifted to the red by 130 cm^−1^ with increasing excitation wavelength from 470 to 540 nm, while in a series of liquids the position of the emission maximum of NR remained constant within same range of the excitation wavelength. It is concluded that the fluorescence spectrum of NR in PMMA undergoes inhomogeneous broadening due to different surroundings of NR molecules in the ground state and slow sub-glass transition (*T*_g_) relaxations in PMMA.

## Introduction

The chain and segment mobility as well as the permittivity of polymers possess an enormous impact on the properties of polymers and polymer thin films. For ultrathin films, in which the film thickness is in the order of the radius of gyration, special effects of confinement have been observed. Prominent examples are the properties of substrate-supported ultrathin polymer films, in which the values of the glass transition temperature (*T*_g_) and segmental mobilities were found to be altered. Likewise, this holds for transport properties, including polymer nanocapsule membrane permeability [1], enzyme-triggered bacterial sensors [2–5] and intelligent self-controlled drug delivery systems [4,6–10], as well as dynamics of polymers at interfaces [11].

**Figure 1:**
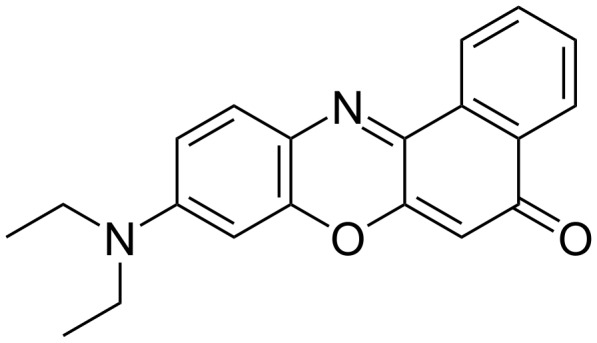
Molecular structure of Nile red (NR).

To be able to understand local properties of polymers, in particular in nanoenvironments of polymeric vesicles (polymersomes), comprising a hydrophilic corona and a hydrophobic wall [1,12], or in substrate-supported ultrathin films [13], the analysis of the photophysical properties of tracer dye molecules was found to be beneficial. In time-resolved fluorescence measurements and dye diffusion studies, the nanoenvironments in polymersomes could be assigned [1,12], solute transport be characterized [1] and segment mobilities inferred [13], respectively. For other purposes the oxazine tracer dye Nile red (NR, Figure 1) served as a local probe to enable the study of degradation of enzyme labile polymersomes [14]. The same dye has been reported as probe for local permittivity in polymers, in particular, the spatial distribution of the dielectric constant measured for thin PMMA films was described using NR as a reported dye [15].

Because NR is (i) a photostable dye, (ii) possesses convenient optical properties, such as excitation with visible light, and fluorescence, which does not overlay with absorption of many biomolecules, and (iii) exhibits fluorescence, which is strongly influenced by the polarity of the environment, it has been employed as a polarity probe in biological applications [16–19] and materials/nanoscience [15,20–23]. For instance, the spatial distribution of the dielectric constant for thin PMMA films was mapped by analyzing the position of the fluorescence of NR employed as a reporter dye [15]. Besides polarity, the Young’s modulus of the polymer matrix was found to be related to the fluorescence lifetimes (τ_f_) of NR [21]. Furthermore, the spectral position of NR fluorescence was used to detect a lipid droplet in monkey aortic smooth muscle cells [16], for visualizing different proteins, such as lactoglobulin, casein and albumin [17]. In fluorescence lifetime imaging microscopy τ_f_ as a viable contrast parameter was employed to image lipid droplets in living HeLa cells stained with NR [19]. Recently, the phase of the microcapsules and their energy release were analyzed by monitoring the color of NR fluorescence in an energy storage microsystem [22].

Although NR solvent effects are a useful tool in biology and technology, an adequate description and characterization of the phenomenon is still far from accurate. Fortunately, a contribution of solute to the large amplitude motion of the diethylamino group (twisting) in intramolecular charge transfer excited state of NR, postulated in references [21,24–25] was later associated with an artefact [26]. However, the characteristics of NR in solvents and in matrices, such as the polarity of NR in the ground and excited states, as one can see in detail below, remains still rather controversial.

Here we aim at the development of a conclusive approach to determine the permittivity of polymers and its spatial distribution as they play a crucial role in the behavior of thin films, as alluded to above. Poly(methyl methacrylate) (PMMA) in submicrometer thin films on a glass support served as a model system.

The relaxation processes in bulk PMMA are well established and have been well characterized by dielectric [27–29] and dynamic mechanical analyses [30–32], NMR spectroscopy [33], and fluorescence spectroscopy [28]. For PMMA, the α-relaxation as slowest relaxation is observed at the glass transition (*T*_g_ = 95–110 °C) [34]. It corresponds to long-range conformational changes of the polymer backbone. This relaxation is frozen in the current experiments. However, the secondary β-, γ-, and δ-relaxations, which correspond to the side chain motions of the ester group and rotations of the methyl groups attached to the main as well as to the side chains, possess characteristic relaxation temperatures *T*_β_ = 10–40 °C, *T*_γ_ = −100 to −170 °C, and *T*_δ_ = −180 °C [28,31]. In PMMA, the dynamics of the ester group (β-relaxation) can furthermore be coupled with the α-relaxation [29].

## Results and Discussion

For the development of a quantitative understanding of the polar probe NR in various nanoenvironments it is imperative to obtain a consistent description of the charge transfer at the electronic transition. For this purpose, the best choice is to study NR in dipolar solvents free from specific interactions with this solute. Finally, the polarity of the polymer matrix that does not possess comparable solute–solvent interactions will be probed with NR.

### Dipole moments

**Figure 2:**
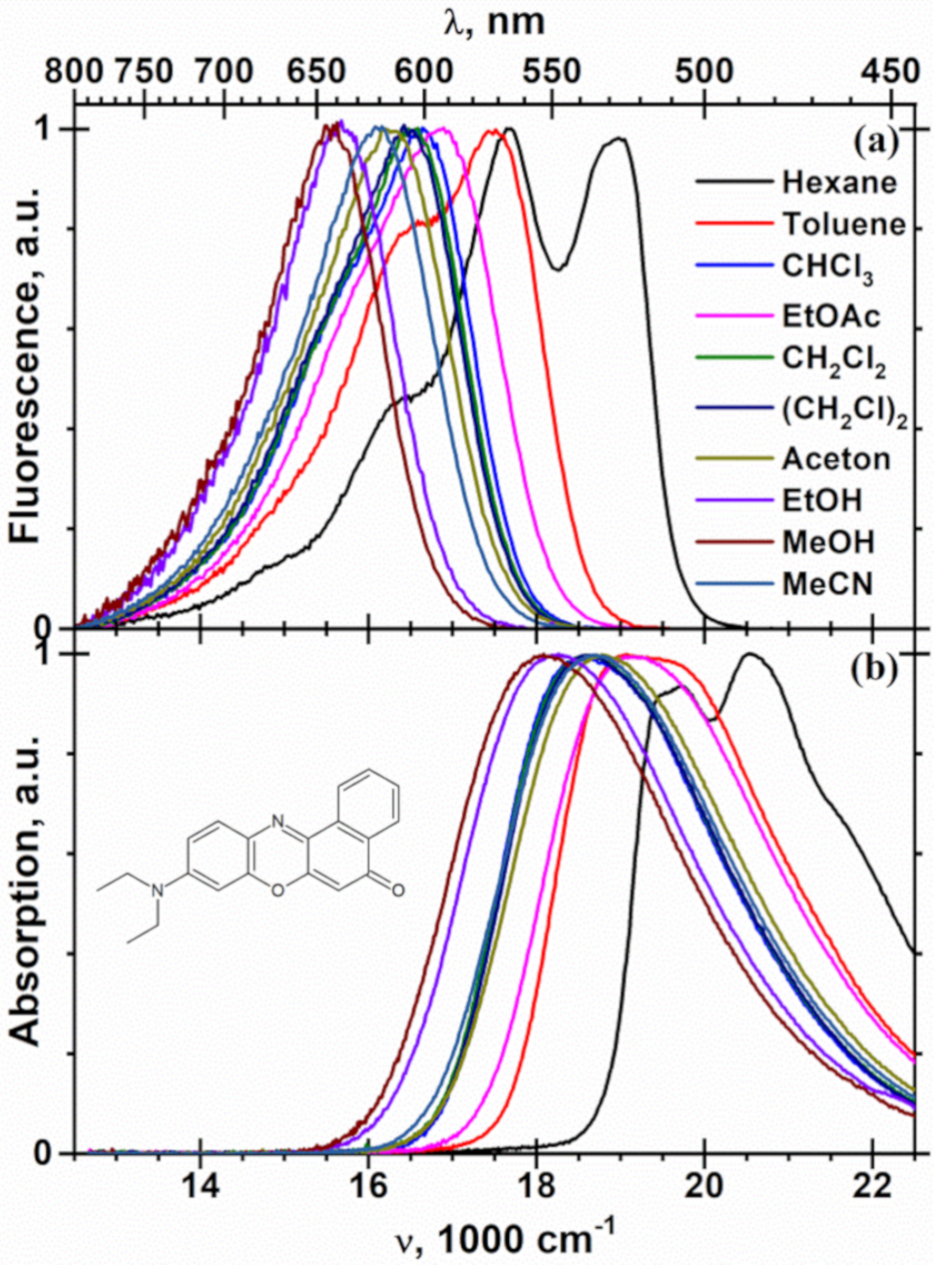
Fluorescence (a) and absorption (b) spectra of NR in solvents of different polarity at 25 °C. The solvent marks are hexane for *n*-hexane, CHCl_3_ for chloroform, EtOAc for ethyl acetate, CH_2_Cl_2_ for dichloromethane, (CH_2_Cl)_2_ for 1,2-dichloroethane, EtOH for ethanol, MeOH for methanol and MeCN for acetonitrile.

With increasing solvent polarity, the maxima in the fluorescence spectra of NR in liquid solvents at 25 °C were observed to gradually shift to the red, from 17660 cm^−1^ in nonpolar *n*-hexane to 16090 cm^−1^ in polar acetonitrile, showing no indication of dual fluorescence (Figure 2a). This behavior for NR [35] is similar to donor–acceptor-substituted stilbenes [36–37], benzenes [38–39] and aminocoumarins [35,40–41]. This effect is caused by solvent relaxation around dipolar solutes that possess in the lowest excited state a substantially higher dipole moment (μ_e_) than that in the ground state (μ_g_).

**Figure 3:**
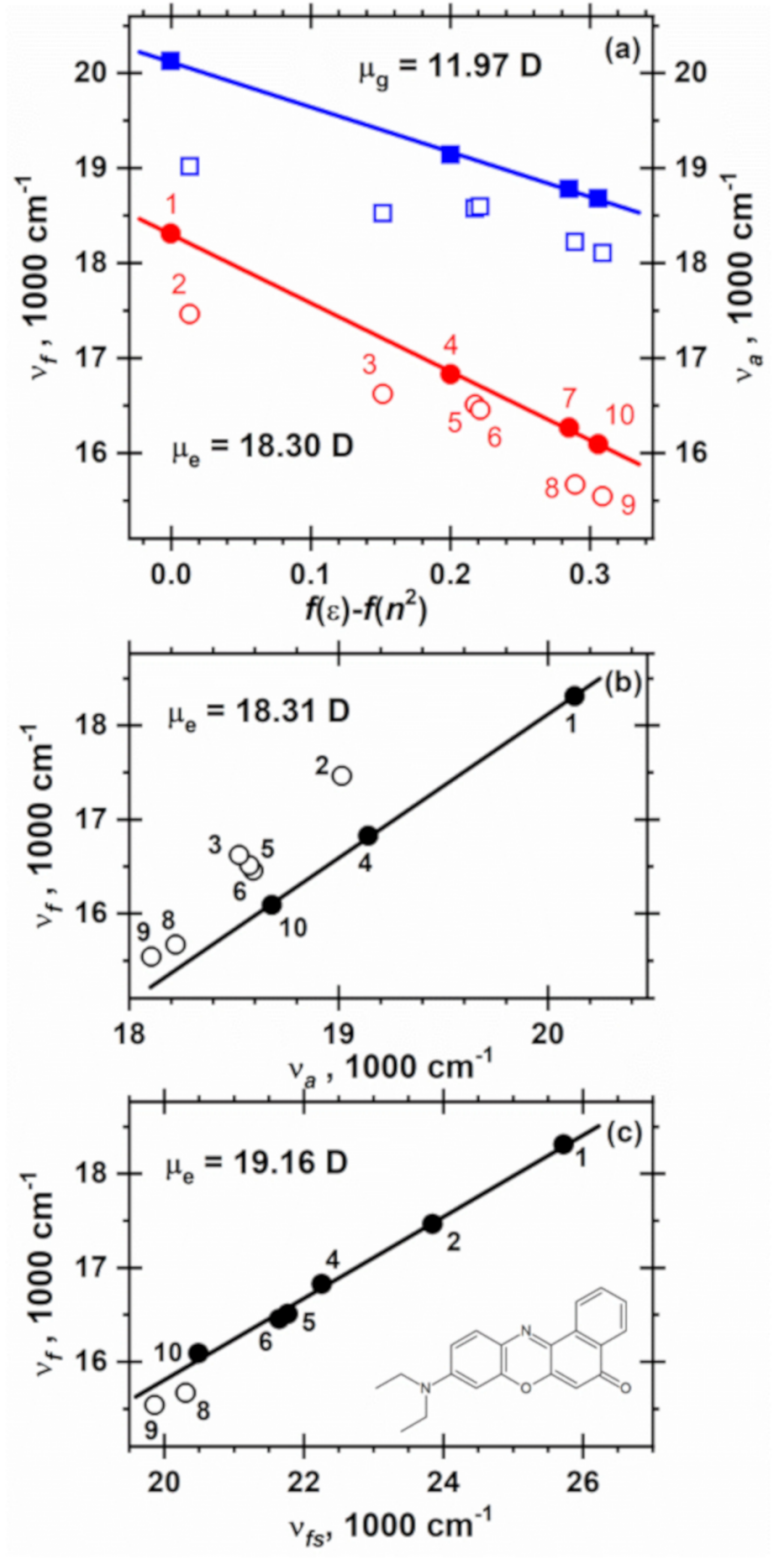
Solvatochromic plot of the absorption (ν_a_) and fluorescence (ν_f_) maxima of NR in a series of solvents at 25 °C (a) against the Lippert solvent polarity function *f*(ε) − *f*(*n*^2^), (b) against absorption maximum ν_a_ and (c) against the fluorescence maximum (ν_fs_) of the intramolecular charge-transfer band of 4-(diisopropylamino)benzonitrile (DIABN). In panel (a) the data for ν_a_ and ν_f_ are shown with blue squares and red circles, respectively. From the slopes of the straight lines fitted through the filled circles and squares according to Equation 1 and Equation 2 in (a), Equation 4 in (b) and Equation 5 in (c) the ground (μ_g_) and excited state dipole moments (μ_e_) of NR are calculated, see Table 2. The solvents are indicated by the numbers in the first column of Table 1.

To calculate the value of μ_e,_ the frequencies of emission (ν_f_) and absorption (ν_a_) are plotted [39,42] in Figure 3a against the Lippert solvent polarity function *f*(ε) − *f*(*n*^2^) [43], where ε is the dielectric constant and *n* is the refractive index of the solvent, *f*(*x*) = (*x* − 1)/(2*x* + 1).

[1] $$\nu_{\text{f}}=-\frac{2\mu_{\text{e}}(\mu_{\text{e}}-\mu_{\text{g}})}{hc\rho^{3}}(f(\varepsilon )-f(n^{2}))+\nu_{0}{}_{\text{f}}$$

[2] $$\nu_{\text{a}}=-\frac{2\mu_{\text{g}}(\mu_{\text{e}}-\mu_{\text{g}})}{hc\rho^{3}}(f(\varepsilon )-f(n^{2}))+\nu_{0}{}_{\text{a}}$$

Here ν_0f_ and ν_0a_ are the frequencies at zero value of the polarity function, ρ is the Onsager cavity radius, *h* is Planck’s constant, and *c* is the speed of light. The experimentally determined positions of the maxima in the absorption and fluorescence spectra of NR as well as the solvent polarity properties are listed in Table 1.

The data points for the dipolar aprotic solvents acetonitrile, acetone and ethyl acetate and the nonpolar *n*-hexane, shown by filled circles in Figure 3a, lie along a straight line. From its slope of −7210 cm^−1^ and μ_g_ = 11.97 D the value of μ_e_ = 18.30 D is calculated. The fluorescence spectra of NR in the protic solvents ethanol and methanol are substantially shifted to the red (Figure 2a), although they possess similar *f*(ε) − *f*(*n*^2^) values compared to those for acetone and acetonitrile, respectively (Table 1). This effect is reminiscent of the anomalous fluorescence red shift of fluorophores in alcohols [44]. The additional red shift of NR in protic solvents, such as alcohols and probably chloroform, is associated with the hydrogen-bond formation with the carbonyl group of the dye [45]. The fluorescence maxima of NR in highly polarizable toluene and in chlorinated solvents lie also clearly below the straight line in Figure 3a. This is tentatively attributed to the different inductive solute–solvent interactions, which are neglected in Equation 1. Such additional red shift in the case of halogenated solvents has been explained before by the formation of exciplexes [46–48].

**Table 1:** Absorption (ν_a_) and fluorescence (ν_f_) maxima of NR, fluorescence maxima (ν_fs_) of the intramolecular charge transfer state of 4-(diisopropylamino)benzonitrile (DIABN) in different solvents, solvent dielectric constants (ε), refractive indexes (*n*) and Lippert polarity functions at 25 °C*.*

N	Solvent	ε^a^	n^b^	*f*(ε) − *f*(*n*^2^)^c^	ν_a_, cm^−1^	ν_f_, cm^−1^	ν_fs_^d^, cm^−1^

1	hexane	1.88	1.372	0.000	20130^d^	17660^d^	25720
2	toluene	2.37	1.494	0.013	19010	17470	23840
3	CHCl_3_^e^	4.89	1.442	0.152	18520	16630	
4	EtOAc^f^	5.99	1.370	0.200	19140	16840	22260
5	CH_2_Cl_2_^g^	8.87	1.421	0.218	18570	16510	21770
6	(CH_2_Cl)_2_^h^	10.36	1.443	0.221	18590	16460	21650
7	acetone	20.56	1.356	0.285	18780	16270	
8	ethanol	24.60	1.360	0.289	18220	15670	20310
9	methanol	32.32	1.327	0.309	18100	15540	19860
10	MeCN^i^	36.65	1.342	0.306	18680	16090	20490

^a^Dielectric constants from ref [49]. ^b^Refractive indexes from ref [50]. ^c^Lippert polarity function *f*(ε) − *f*(n^2^). ^d^Half-sum of wavenumbers for the maxima of the first and second vibronic peaks in the spectrum possessing practically equal intensities, see Figure 1. ^e^Chloroform. ^f^Ethyl acetate. ^g^Dichloromethane. ^h^1,2-Dichloroethane. ^i^Acetonitrile.

In polar solvents the absorption band of NR is shifted to the red, from 19670 cm^−1^ in *n*-hexane down to 18680 cm^−1^ in acetonitrile (Figure 3a), in the same direction as its fluorescence spectrum. A somewhat smaller magnitude of the shift of 990 cm^−1^ compared to that observed in the fluorescence spectrum (1570 cm^−1^) indicates a higher dipole moment in the excited state (μ_e_ > μ_g_) and a larger negative slope in Equation 1 ~μ_e_(μ_e_ − μ_e_) than in Equation 2 ~μ_g_(μ_e_ − μ_g_). This solvatochromic plot with its slope of −4730 cm^−1^ resembles plot according to Equation 1 in Figure 3a, namely the points for dipolar acetonitrile, acetone, ethyl acetate and nonpolar *n*-hexane fit to a straight line (2), NR in other solvents exhibits again a stronger red shift due to specific and inductive solute–solvent interactions.

In order to eliminate any scaling effect of the Onsager radius on the relation between μ_g_ and μ_e_, Equation 2 can be rearranged to obtain the polarity function

[3] $$f(\varepsilon )-f(n^{2})=\frac{hc\rho^{3}}{2\mu_{\text{g}}(\mu_{\text{e}}-\mu_{\text{g}})}(\nu_{0}{}_{\text{a}}-\nu_{\text{a}}).$$

Substitution of Equation 3 in Equation 1, assuming equal ρ values of the same molecule for the absorption and emission transitions, gives the following simple Onsager radius-independent linear relation between emission and absorption frequencies.

[4] $$\nu_{\text{f}}=\frac{\mu_{\text{e}}}{\mu_{\text{g}}}\nu_{\text{a}}+\nu_{0}{}_{\text{f}}-\frac{\mu_{\text{e}}}{\mu_{\text{g}}}\nu_{0}{}_{\text{a}}$$

In Figure 3b the fluorescence maxima of NR are plotted versus the absorption maxima. The solvent is indicated by its number according to Table 1. Data for solvents that possess similar refractive indices (*n* = 1.342−1.347) fit to Equation 4 with a slope 1.529, indicating a substantial (53%) increase of the dipole moment upon excitation of NR. A clear deviation is observed for protic solvents and highly polarizable solvents (with *n* = 1.421−1.494) due to the different efficiency of hydrogen bond formation and contributions of inductive solute–solvent interactions in the ground and excited states.

The slopes of the two linear relations Equation 1 α = −2μ_e_(μ_e_ − μ_g_)/*hc*ρ^3^ and Equation 4 β_a_ = μ_g_/μ allow one to calculate both ground and excited states dipole moments as

[6] $$\mu_{\text{g}}=\sqrt{-\frac{\alpha hc\rho^{3}}{2\beta_{\text{a}}(\beta_{\text{a}}-1)}}$$

[7] $$\mu_{\text{e}}=\sqrt{-\frac{\alpha \beta_{\text{a}}hc\rho^{3}}{2(\beta_{\text{a}}-1)}}$$

From α = −7210 cm^−1^ (Figure 3a) and the β_a_ = 1.529 (Figure 3b), one obtains μ_g_ = 11.97 ± 0.36 D and μ_e_ = 18.30 ± 0.61 D, see Table 2.

**Table 2:** Ground (μ_g_) and excited (μ_e_) state dipole moments of NR.

Equation	Slope, cm^−1^	μ_g_^a^, D	ρ, pm	μ_e_^b^, D

1	−7210 ± 110	11.97± 0.35	545	18.30 ± 0.53
2	−4730 ± 100	11.97± 0.35	545	18.31 ± 0.47
4	1.529^c^ ± 0.023^c^	11.97± 0.35	545	18.30 ± 0.61
8	0.434^c^ ± 0.015^c^	11.97± 0.35 6.78^d^	545 468^d^	19.16 ± 0.19 18^d^
2^e^	−6000 ± 420	11.97± 0.35	545	20.00 ± 0.51
10^e^	0.366^c^ ± 0.019^c^	11.97± 0.35 6.78^d^	545 468^d^	21.68 ± 0.51 18^d^

^a^Other reported values (in Debye units D) are 7 [47,51–52], 7 [53–54] (for phenoxazone 9, the *N*,*N*-dimethylamino analogue of NR), 7.51 [55], 7.97 [55], 8.2 [56–57], 8.4 [44], 8.9 [44], 14 [48]. ^b^Other values (in D) 6.9 [52], 7.7 [51], 8.5 [51], 10.0 [57], 10.2 [56], 10.4 [58], 10.5 [58], 10.77 [55], 12.48 [55], 13.15 [55], 13.4 [44], 14.13 [55], 14.4 [44], 14.5 [47], 17 [47], 18 [48], 18 [53–54] (for phenoxazone 9, the *N*,*N*-dimethylamino analogue of NR), 18.5 [48], and 18.6 [51–52]. ^c^Dimensionless. ^d^Values for DIABN [38–39]. ^e^Absorption maxima from [59].

Using a similar method [60–61], in which it is not necessary to assume the radius ρ, the ratio of the slopes of the solvatochromic plots for fluorescence (Equation 1) and absorption (Eqution 2) also yields a value for β_a_. Although closely matching values of β_a_ are expected for both the ratio method and from the direct correlation according to Equation 4, when the same set of solvents is employed in Equations 1, 2 and 4, the resulting values of β_a_ could be erroneous, if *different* sets of solvents were used in Equation 1 and Equation 2.

Alike to Equation 4, the excited state dipole moment can be determined from a plot of the fluorescence maxima of the studied fluorophore versus the fluorescence maxima of a standard fluorophore with known ground and excited dipole moments μ_gs_ and μ_es_ [38–39]. Application of Equation 1 to the spectra of the standard fluorophore and rearrangement in order to express solvent polarity function yields

[8] $$f(\varepsilon )-f(n^{2})=\frac{hc\rho_{\text{s}}{}^{3}}{2\mu_{\text{es}}(\mu_{\text{es}}-\mu_{\text{gs}})}(\nu_{0}{}_{\text{f}s}-\nu_{\text{fs}}).$$

Then substitution of the solvent polarity function again into Equation 1 for the studied fluorophore gives a linear correlation (Equation 9) of ν_f_ and ν_fs_

[9] $$\nu_{\text{f}}=\frac{\mu_{\text{e}}(\mu_{\text{e}}-\mu_{\text{g}})\rho_{\text{s}}{}^{3}}{\mu_{\text{es}}(\mu_{\text{es}}-\mu_{\text{gs}})\rho^{3}}\nu_{\text{fs}}+\nu_{0}{}_{\text{f}}-\frac{\mu_{\text{e}}(\mu_{\text{e}}-\mu_{\text{g}})\rho_{\text{s}}{}^{3}}{\mu_{\text{es}}(\mu_{\text{es}}-\mu_{\text{gs}})\rho^{3}}\nu_{\text{0fs}}.$$

The subscript *s* indicates values related to the above mentioned standard. In Equation 9 the effect of the very important parameter, the Onsager radius, on the resulting dipole moment also decreases, because the ratio ρ_s_^3^/ρ^3^ should be practically independent from the method how these radii are estimated.

In Figure 2c the fluorescence maximum data are plotted versus that of the intramolecular charge transfer state of 4-(diisopropylamino)benzonitrile (DIABN). Data for all solvents except for the alcohols are fitted well to the linear Equation 9 with a positive slope β_f_ = 0.434. The value

[10] $$\mu_{\text{e}}=\frac{\mu_{\text{g}}}{2}+\sqrt{\frac{\mu_{\text{g}}{}^{2}}{4}+\beta_{\text{f}}\mu_{\text{es}}(\mu_{\text{es}}-\mu_{\text{gs}})\frac{\rho^{3}}{\rho_{\text{s}}{}^{3}}}$$

of 19.16 ± 0.19 D agrees well with that determined from the solvatochromic expressions (Equations 1, 2 and 4), see Table 2.

Substitution of the polarity function (Equation 8), calculated with the reference fluorophore in Equation 2, gives the following relations between absorption maxima of NR and emission maxima of the dipole moment standard:

[5] $$\nu_{\text{a}}=\frac{\mu_{\text{g}}(\mu_{\text{e}}-\mu_{\text{g}})\rho_{\text{s}}{}^{3}}{\mu_{\text{es}}(\mu_{\text{es}}-\mu_{\text{gs}})\rho^{3}}\nu_{\text{fs}}+\nu_{0}{}_{\text{a}}-\frac{\mu_{\text{g}}(\mu_{\text{e}}-\mu_{\text{g}})\rho_{\text{s}}{}^{3}}{\mu_{\text{es}}(\mu_{\text{es}}-\mu_{\text{gs}})\rho^{3}}\nu_{\text{0fs}}.$$

With the slope of the linear function (10)

[11] $$\beta_{\text{f}}=\frac{\mu_{\text{g}}(\mu_{\text{e}}-\mu_{\text{g}})\rho_{\text{s}}{}^{3}}{\mu_{\text{es}}(\mu_{\text{es}}-\mu_{\text{gs}})\rho^{3}}.$$

The excited state dipole moment can be evaluated as

[12] $$\mu_{\text{e}}=\mu_{\text{g}}+\beta_{\text{f}}\frac{\mu_{\text{es}}(\mu_{\text{es}}-\mu_{\text{gs}})\rho^{3}}{\mu_{g}\rho_{\text{s}}{}^{3}}.$$

The published values of the dipole moments [44,47–48,51–56] of NR, summarized in the footnote of Table 2, are almost uniformly dispersed in the wide range from 7 to 14 D for μ_g_ and from 6.9 to 19.6 D for μ_e_, respectively. All data demonstrate an increasing of dipole moment at the excitation μ_e_ − μ_g_ between 1.8 and 11.6 D [44,51,56,62–63], also with μ_g_ = 7 D and μ_e_ = 6.9 ± 2.1 D [52] it is still correct within the experimental uncertainty. Thus, the values of NR μ_g_ = 11.97 D and μ_e_ − μ_g_ from 6.34 to 7.20 D obtained here (Table 2) agree with the published data. The excited state dipole moment μ_e_ from 18.30 to 19.16 D and the slightly higher μ_e_ = 20–22 D (Figure S1, Supporting Information File 1, Table 2) calculated from the published absorption maxima of NR [59] using Equation 2 and Equation 5 is on the upper bound of the published μ_e_ values.

From a theoretical point of view, the very broad distribution of dipole moments published in the literature [44,47–48,51–58] might be caused because (i) various experimental approaches were used, including solvatochromic, thermochromic and dielectric friction techniques, and (ii) different theoretical models of solvatochromic effects were employed, some of them take inductive solute–solvent interactions and solute polarizability into account, others neglect them, and (iii) different definitions of the molecular dipole moment in solvent-free conditions in vacuum or in solvents with zero polarity function *f*(ε) − *f*(n^2^) were used. Most of these divergences can be compensated by an appropriate selection of the solvent set. For example, the effect of inductive solute–solvent interactions on ν_a_ or ν_f_ becomes practically invariant, when the refractive index is constant in a series of solvents. From an experimental point of view, the broad distribution of reported dipole moments could also be the result of an imprecise correction of emission spectra for the spectral response or their imprecise presentation on the wavenumber scale. The major challenge appears to be the lack of independently determined parameters of the NR ground state dipole moment and/or its cavity radius, which are both important.

Two values of the Onsager radius, 410 pm [51–52,55,57–58] and 500 pm [44,47,57,62], were used in [44,47–48,51–56,62–63] to evaluate μ_e_ or μ_e_ − μ_g_. The first value is the van der Waals radius ρ = (3*V*/4π)^1/3^, where the van der Waals volume *V* is the sum of atomic increments [64]. The second value was chosen arbitrarily as a typical value. In addition, both these values of ρ were not verified with respect to their applicability for use in solvatochromy of NR.

In the present paper, we used ρ = ρ_0_(*M*/*M*_0_)^1/3^, where *M* is the molar mass of the fluorophore and the lower index ‘0’ marks the corresponding quantity related to *N*,*N*-dimethylaminobenzonitrile (DMABN). The Onsager radius of DMABN was tailored to ρ_0_ = 420 pm [39] in order to get μ_e_ = 17 D for the charge transfer excited state of DMABN from the solvatochromic plot (Equation 1). The corresponding dipole moments were determined with cavity radius free techniques: μ_g_ = 6.60 D [39] by dielectric spectroscopy and μ_e_ = 17 D [65] by time-resolved microwave conductivity. For the secondary standard DIABN ρ_s_ = 468 pm is calculated from ρ_0_, μ_gs_ = 6.78 was measured by dielectric spectroscopy and μ_gs_ = 18 D was determined from the solvatochromic plot (Equation 9) relative to the wavenumber maxima of the excited state charge transfer emission spectra of DMABN [38–39]. For NR a cavity radius of ρ = 545 pm is calculated based on ρ_0_ and *M*.

When the literature values of the absorption maxima of NR [59], ρ = 545 pm and μ_g_ = 11.97 D are fitted with Equation 2 and Equation 5, values that are close to the values of μ_e_ = 20–22 D reported here are obtained (Table 2). Because the cavity radius for NR is adapted for solvatochromy in our work, reliable values of the ground state and excited state dipole moments of NR can indeed be calculated.

The crucial importance of the correct value for the Onsager radius is demonstrated by fitting data from Table 1 with Equation 2 and Equation 6 for a van der Waals radius ρ = 410 [51–52,55,57–58]. This fit yields μ_g_ = 7.81 D instead of 11.97 D (Table 2) and μ_e_ = 11.94 D instead of 18.30 D (Table 2). These values are close to μ_g_ = 8.2 D and μ_e_ = 14.4 D from reference [56], where both dipole moments were determined by similar analysis of absorption and fluorescence spectra of NR. Thus, the large dispersion of reported NR dipole moments [44,47–48,51–56,62–63] is mainly caused by insufficient precision of the value for the cavity radius of NR.

### Polymer polarity probed with NR

**Figure 4:**
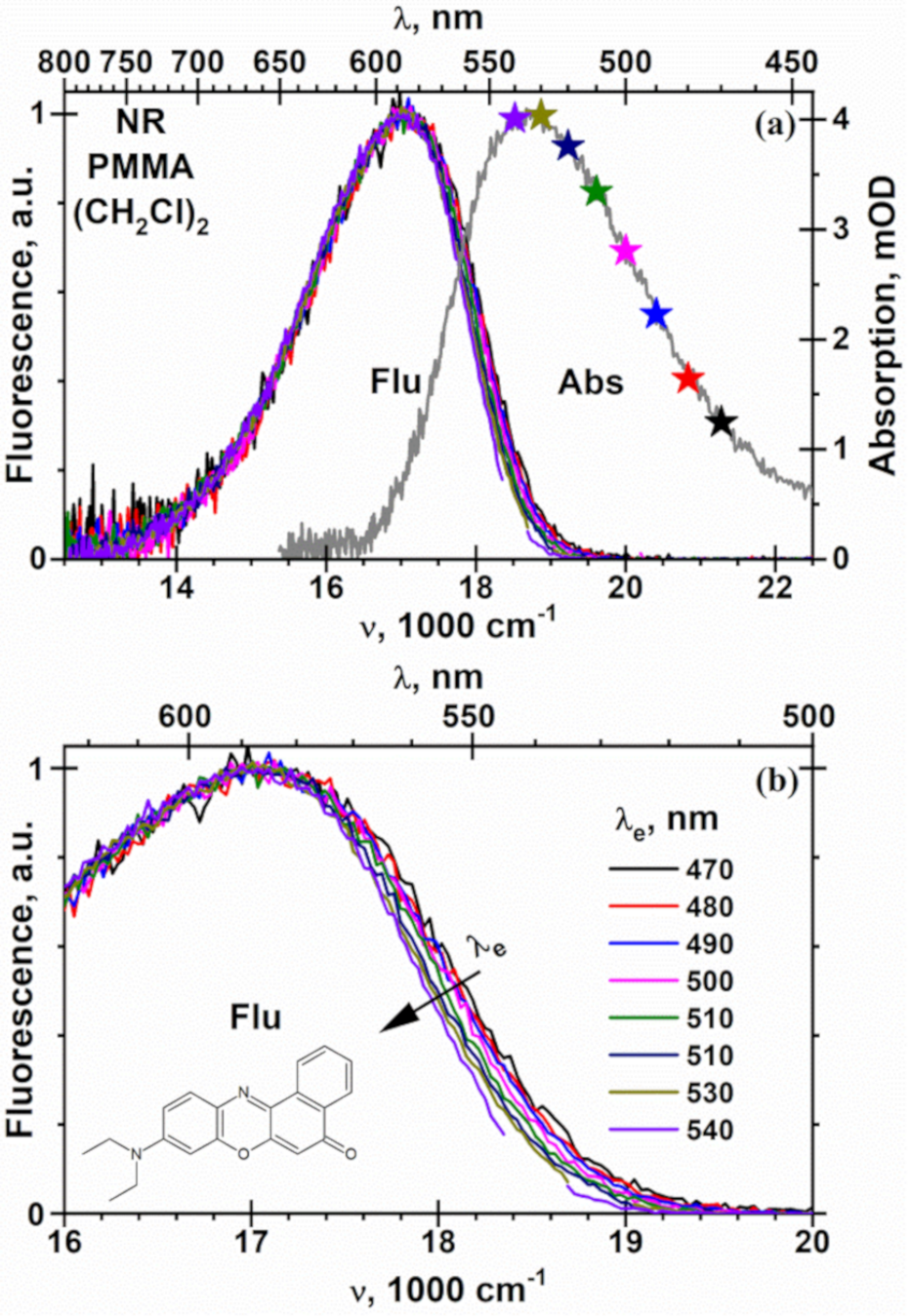
Absorption (a) and fluorescence (a, b) spectra of NR in PMMA (350 kg/mol) film 500 nm thin at different excitation wavelength (λ_e_). The film was prepared by spin-coating of PMMA solution in 1,2-dichloroethane ((CH_2_Cl)_2_) on the 20 × 20 × 0.15 mm^3^ glass doped with NR. The concentration of NR in the PMMA film of 2.1 mM was calculated from the optical density (panel (a)) and thickness of PMMA film with the molar extinction coefficient NR in 1,4-dioxane of 38000 M^−1^·cm^−1^ [68]. The λ_e_ values are indicated with stars on the absorption spectrum in panel (a). The regions λ_e_ ± 5 nm in the fluorescence spectra (a, b) are not shown due to overlap with strong scattering excitation light.

The fluorescence spectra of NR in low and high molar mass PMMA films (Figure 4, Figure S2, Supporting Information File 1) were found to be close to spectra acquired in ethyl acetate (Figure 2a), which may be used as a model for the polymer repeat unit. The broad structureless fluorescence maximum at ≈600 nm for NR in PMMA reported in references [62,66] might be caused by remaining traces of solvent from PMMA solution (toluene) [66] in close vicinity to NR molecules, similar to results for NR in poly(vinylidene fluoride) films cast from dimethyl sulfoxide [15]. This notion is supported by fluorescence maxima (λ_f_) of NR in PMMA at the excitation wavelength λ_e_ = 500 nm, which are in the range of 571 < λ_f_ < 579 nm, depending on the polymer molar mass and casting solvents. For ethyl acetate, the fluorescence of NR occurs at longer wavelength λ_f_ = 588 nm, which does not agree with the results in reference [66].

**Figure 5:**
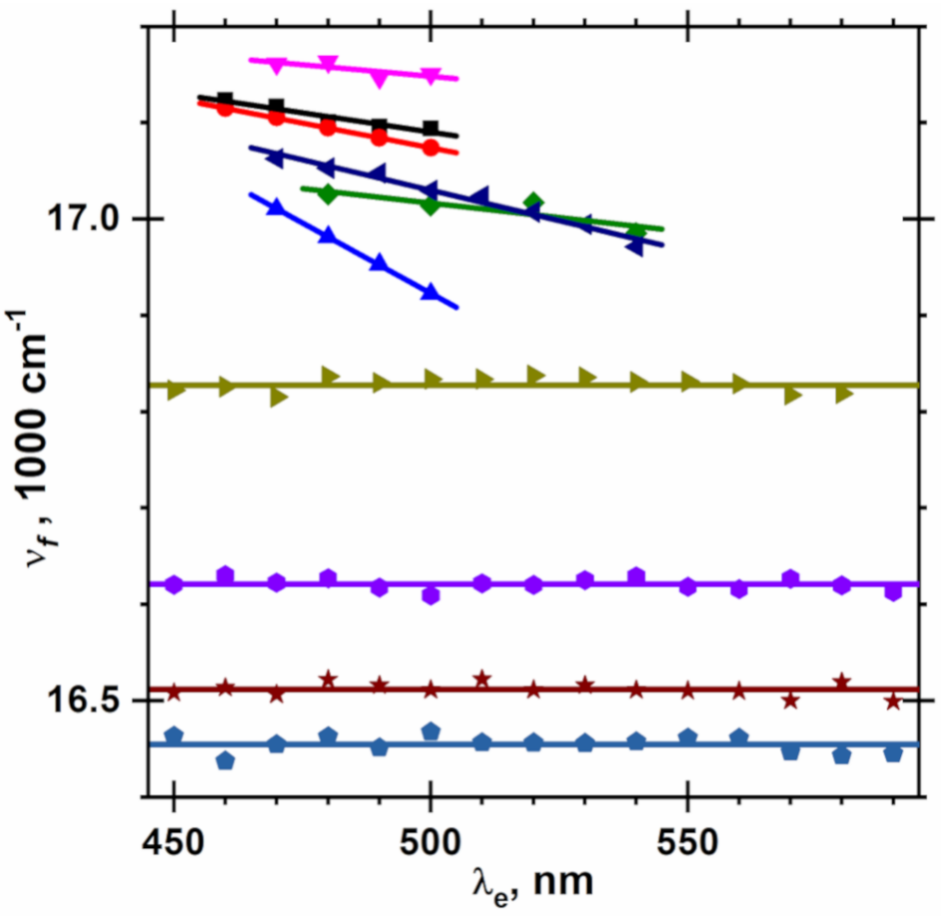
Dependence of the fluorescence maximum (ν_f_) of NR on the excitation wavelength (λ_e_) in rigid PMMA matrix (six data sets on the top) and in fluid solutions (four data sets on the bottom) at 25 °C. The details of the preparation of the submicron PMMA films are listed in Table 3. The straight lines for NR in the PMMA matrices and horizontal lines for NR fluid solutions at λ_e_ = 500 nm are shown from top to bottom in the following series: PMMAH from CH_2_Cl_2_, PMMAL from CH_2_Cl_2_, CHCl_3_ and (CH_2_Cl)_2_, PMMAH from CHCl_3_ and (CH_2_Cl)_2_, ethyl acetate, CHCl_3_, CH_2_Cl_2_, and (CH_2_Cl)_2_, respectively.

**Figure 6:**
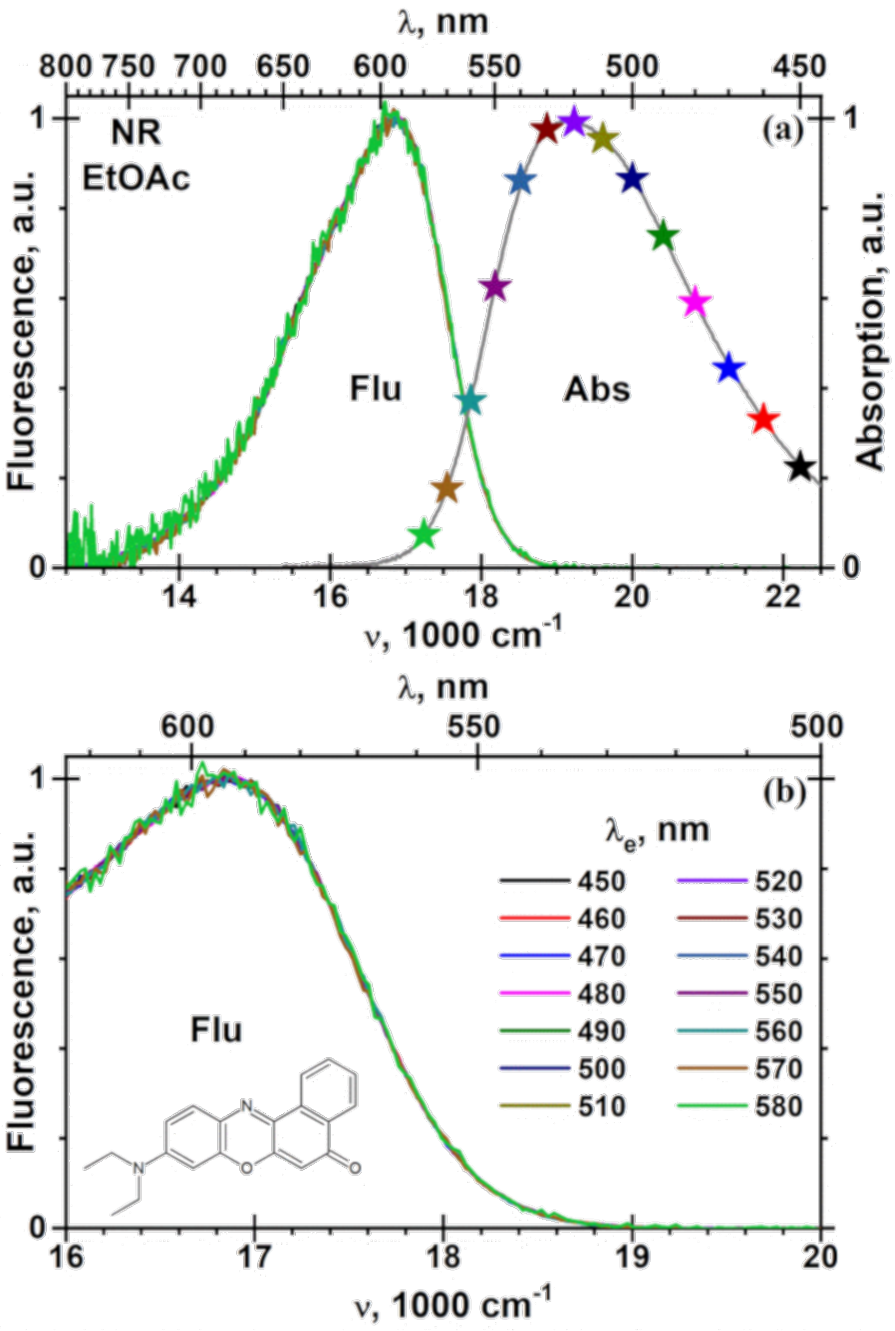
Absorption (a) and fluorescence (a, b) spectra of NR in ethyl acetate (EtOAc) at different excitation wavelength (λ_e_) at 25 °C. The λ_e_ values are indicated with the stars on the absorption spectrum in panel (a).

When λ_e_ increases, the fluorescence spectrum of NR in the polymer matrix shifts linearly to the red by 130 cm^−1^ from 17100 at λ_e_ = 470 nm to 16970 cm^−1^ at λ_e_ = 540 nm, mainly in the blue part of the spectrum and at the maximum (Figure 4, Figure 5 and Figure S2, Supporting Information File 1). In contrast to PMMA, neither the shape of the fluorescence spectrum of NR nor the position of its maximum depend on λ_e_ in liquid solutions, such as in the polymer repeat unit model ethyl acetate, and in solvents used for preparation of spin-coated PMMA films, such as chloroform, dichloromethane and 1,2-dichloroethane (Figure 5, Figure 6 and Figures S3, S4 and S5, Supporting Information File 1). These facts and the blue shifted fluorescence of NR in PMMA in comparison to mentioned above solutions (Figure 5) demonstrate that the local polarity in PMMA and/or its dynamics are different than in similar liquid solutions. The fluorescence maxima of NR at λ_e_ = 500 nm, the slopes *d*ν_f_/*d*λ_e_ of the fluorescence spectrum drift with the excitation wavelength and the thicknesses of spin-coated PMMA films are collected in Table 3. On the one hand, such independence of the fluorescence spectra of NR in liquid solution supports the high purity of the NR used and its high stability also in the chlorinated solvents, cf. with a lack of a red edge effect for 4-fluoro-*N*,*N*-dimethylaniline [67]. On the other hand, when the relaxation time of the molecules or segments (τ_r_) of the polymer matrix is much longer than the NR fluorescence decay time τ_f_ = 3.87 ns [62] in PMMA and of ≈5 ns [62] in the liquid solvents used, the orientational relaxation of the molecules or segments around in the excited NR is not complete. Consequently, the fluorescence of NR in polymers originating from a not solvent-relaxed excited state possesses higher energy. The corresponding matrix polarity is characterized by an effective dielectric constant ε(2π/τ_f_) between ε, the zero frequency, and ε_∞_ = *n*^2^, the optical frequency permittivity of the medium.

[13] $$\varepsilon \left(\frac{2\pi }{\tau_{\text{f}}}\right)=\varepsilon_{\infty }+\frac{\varepsilon -\varepsilon_{\infty }}{1+{\left(\frac{\tau_{\text{r}}}{\tau_{\text{f}}}\right)}^{2}}$$

**Table 3:** Fluorescence maxima (ν_f_) of NR at the excitation wavelength (λ_e_) of 500 nm in PMMA spin-coated film of thickness (*d*), ν_f_ drift rate (*dν*_f_/*d*λ_e_)*,* Lippert solvent polarity function and dielectric constant (ε) of the PMMA films at 25 °C*.*

PMMA^a^	Solvent	ν_f_, cm^−1^	−*dν*_f_/*d*λ_e_ µm^−2^	*d,* nm	*f*(ε) − *f*(*n*^2^)^b^	ε^c^

PMMA-L	CH_2_Cl_2_^d^	17120	8	400	0.167 ± 0.004	6.4 ± 0.3
	CHCl_3_^e^	17070	10	480	0.170 ± 0.004	6.6 ± 0.3
	(CH_2_Cl)_2_^f^	16920	29	380	0.191 ± 0.005	8.3 ± 0.5

PMMA-H	CH_2_Cl_2_^d^	17150	5	960	0.159 ± 0.004	5.9 ± 0.2
	CHCl_3_^e^	17020	6	480	0.178 ± 0.005	7.2 ± 0.4
	(CH_2_Cl)_2_^f^	17040	13	500	0.176 ± 0.005	7.0 ± 0.3

^a^Average molar masses are 33 kg/mol (PMMA-L) and 350 kg/mol (PMMA-H). ^b^Estimated from Equation 10 for NR using slope from Table 2 and intercept ν_0f_ = 18300 ± 26 cm^−1^ for the straight line (Equation 1) in Figure 3a. ^c^Calculated from PMMA Lippert solvent polarity function *f*(ε) − *f*(*n*^2^) and *n* = 1.490 [70]. ^d^Dichloromethane. ^e^Chloroform. ^f^1,2-Dichloroethane.

In a similar manner, the dipolar units are dispersed around NR molecules in the ground state. The observable absorption spectrum is a superposition of the spectra of such partial solvates of NR. According to Equation 2 the absorption of stronger solvated NR molecules is shifted to the red. The more NR is irradiated in the red, the higher is the contribution of stronger solvated NR molecules that are excited. A response time τ_r_ of seconds for a polarization dynamics was estimated for PMMA films in an external electric field [69]. Although short τ_f_ << τ_r_ in the region of 2π/τ_f_ ≈ 1 GHz prevent a substantial rearrange of the solvation shell around the NR molecules in the excited state, solvated molecules absorbing more in the red also fluoresce in the red tail of the spectrum. Such inhomogeneous broadening of absorption and fluorescence spectra can explain the observable drift in the fluorescence spectra of NR in PMMA to the red for long wavelength excitation (Figure 4 and Figure S2, Supporting Information File 1).

The value of νf decreases in the series of the spin-coating solvents dichloromethane, chloroform and 1,2-dichloroethane. The absolute value of the red drift rate *dν*_f_/*d*λ_e_ gradually grows up in the same series. This cast solvent effect of 200 and 130 cm^−1^ with low and high PMMA molar mass is more pronounced than the influence of PMMA molar mass in the same solvent, which were 30, 50 and 120 cm^−1^ for dichloromethane, chloroform and 1,2-dichloroethane, respectively. These results for ν_f_ and −*dν_f_*/*d*λ*_e_* can be rationalized by correlating them with the boiling points of these solvents of 34.6 °C, 62.2 °C and 83.5 °C, respectively. Due to fast solvent evaporation of the most volatile solvent, i.e., dichloromethane, the polymer units are less ordered or dense around the NR molecules. For the least volatile solvent, i.e., 1,2-dichloroethane, a more ordered surrounding of the NR molecules in the ground state leads to a red shift of the fluorescence spectra. The observation might also be explained by a smaller residual amount of solvent for the volatile dichloromethane than for 1,2-dichloroethane and a corresponding plasticizing effect.

The effective Lippert polarity function is determined from the linear Equation 1 by using ν_f_ of NR in PMMA measured at λ_e_ = 500 nm, where an overlap of the fluorescence spectra with scattered excitation light is minimal (Figure 4 and Figure S2, Supporting Information File 1). In parallel to ν_f_, the polarity function, having values between 0.159 and 0.191 close to that of the model solvent ethyl acetate (0.200, Table 3), depends more sensitively on the spin-coating solvent than on the molar mass of the PMMA. Based on the magnitudes of the refractive index of PMMA, *n* = 1.490 [70], 1.491 [71] at 578.1 nm and 1.4868 [72] at 600 nm, the effective local dielectric constants are estimated as 5.9–8.3, which agrees with published data [70,73–74]. The value of ε(2π/τ_f_) in Equation 13 clearly exceeds the high frequency permittivity of PMMA (*n*^2^ = 2.22), indicating substantial mobility of the local environment of NR in the PMMA matrix. For bulk PMMA, dielectric spectroscopy measurements gave values for ε = 3.7 [70], 4.99 [73] and 8 [74] for PMMA films. In reference [15] the local polarity of PMMA film probe by NR was found ε = 3.64. This value is probably underestimated, because in contrast to the present study λ_f_ of NR was estimated not from the spectrum, but from the ratio of integrated fluorescence intensities above and below ≈600 nm. In the following analysis ε was evaluated directly from a double-exponential fit of ε(λ_f_) without any correction for *n*, which would have been necessary.

## Conclusion

New types of relative solvatochromic plots, in which the position of the emission maximum is plotted versus the position of absorption maximum or vice versa, allow one to estimate the ratio of the ground and excited state dipole moments. The values obtained are practically independent from the magnitude of the Onsager cavity radius. The absorption and fluorescence spectra of NR shift to the red with increasing solvent polarity, because of the intramolecular charge transfer character of the optical transition. From the plots of the maxima of the fluorescence and absorption wavenumber spectra in dipolar solvents, which possess negligible specific solute–solvent interactions, versus the Lippert solvent polarity function, and from this fluorescence frequency against corresponding absorption frequency and from the fluorescence maxima of DIABN with known dipole moments, consistent values of the ground and excited state dipole moments of 11.97 D and 18.30–19.16 D were calculated for NR using an Onsager radius of 545 pm. The local environment of NR as local polarity probe molecule in PMMA films demonstrates a local mobility that is higher than that expected from the permittivity of bulk PMMA. The local dielectric constant of 5.9–8.3 implies that beside electronic and atomic polarization (rearrangement of atomic bonds and valence angles) a certain dipolar orientation degree of freedom take place in PMMA within the lifetime of excited state of NR. The restriction of the orientational relaxation causes an inhomogeneous broadening of the fluorescence spectrum of NR and its excitation wavelength dependence. The inhomogeneity depends stronger on the condition of PMMA film preparation than on the PMMA molar mass.

## Experimental

**General**. NR was purchased from Carl Roth (Germany) and was used as received. Poly(methyl methacrylate) (PMMA) with an average molar mass of 350 kg/mol and 33 kg/mol was used (Aldrich, USA). Uvasol acetone, *n*-hexane and methanol (Merck, Germany), absolute ethanol and chloroform (VWR, Germany), 1,2-dichloroethane (Carl Roth, Germany), toluene, dichloromethane, acetonitrile and ethyl acetate (Fisher Scientific, Germany) were used without further purification. The solvents were checked for the lack of fluorescence when excited with 450–600 nm. Polymer films with or without NR were prepared by spin-casting of 3 wt % polymer solutions in 1,2-dichloroethane, dichloromethane or chloroform on silicon wafers (for ellipsometry) and on 20 × 20 × 0.15 mm^3^ Metzler glass coverslip (for optical measurement) at 1000 rpm for 25 seconds using a homebuilt spin-coater. NR stock solutions in the same solvent were added to the polymer solution to get NR-labeled PMMA films. After the spin-coating the samples were kept in the fume hood overnight for evaporation of the solvents.

**Measurements** Absorption and fluorescence spectra were measured at 25 °C by Cary 50 and Cary Eclipse (Varian, Australia) spectrometers, respectively. The fluorescence spectra were corrected for the spectral response. The thickness of the PMMA films were measured with an alpha-SE ellipsometric (J.A. Woollam Co., USA) according to published protocols [2–3].

## Supporting Information

File 1 Solvatochromic plots and fluorescence spectra of NR.
