# Supplementary material for: Probing of local polarity in poly(methyl methacrylate) with the charge transfer transition in Nile red
Source: Beilstein J Org Chem. 2019 Oct 25;15:2552–62. doi: 10.3762/bjoc.15.248 (PMC6839560; doi:10.3762/bjoc.15.248)
Supplement: File 1 — Solvatochromic plots and fluorescence spectra of NR. [file Beilstein_J_Org_Chem-15-2552-s001.pdf]

BEILSTEIN JOURNAL OF ORGANIC CHEMISTRY

Supporting Information

for

Probing of local polarity in poly(methyl methacrylate) with the charge transfer transition in Nile red

Aydan Yadigarli, Qimeng Song, Sergey I. Druzhinin and Holger Schönherr

Beilstein J. Org. Chem. **2019,** *15,* 2552–2562. doi:10.3762/bjoc.15.248

Solvatochromic plots and fluorescence spectra of NR

License and Terms: This is a supporting information file under the terms of the Creative Commons Attribution License (http://creativecommons.org/licenses/by/4.0). Please note that the reuse, redistribution and reproduction in particular requires that the authors and source are credited.
The license is subject to the *Beilstein Journal of Organic Chemistry* terms and conditions: (https://www.beilstein-journals.org/bjoc)

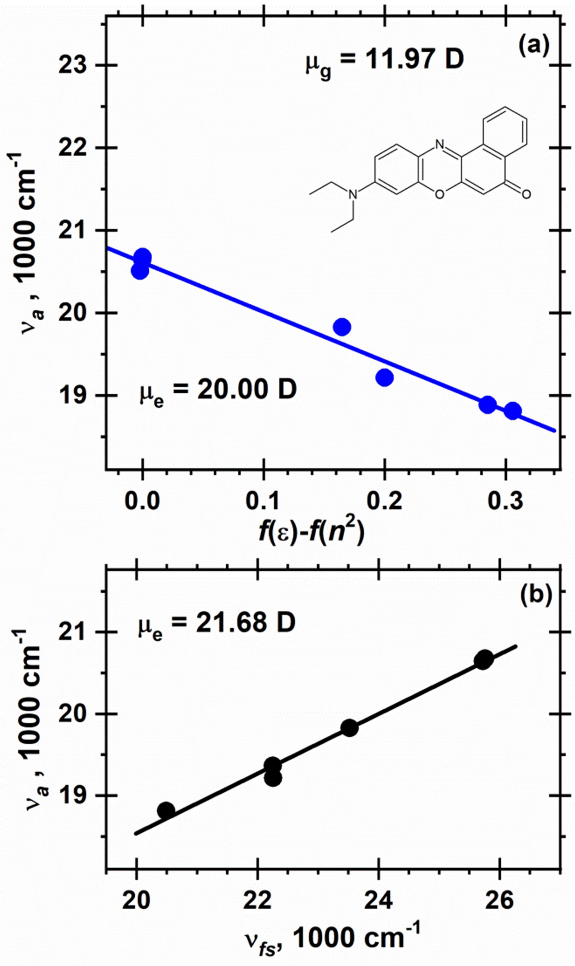

Figure S1: Solvatochromic plots of the absorption maxima (ν_a) of Nile red (NR) in a series of solvents against the Lippert solvent polarity function $f(\varepsilon) - f(n^2)$ at 25 °C (a) and against the fluorescence maxima (ν_{fs}) of the intramolecular charge-transfer band of 4-(diisopropylamino)benzonitrile (DIABN) at 25 °C. From the straight lines corresponding to equation (1) in panel (a) and equation (10) in panel (b) the excited state dipole moments μ_e of 20.00 ± 0.51 D (a) and of 20.68 ± 0.51 D (b) are calculated, see Table 2. The experimental data for absorption maxima of Nile red were taken from [1]. The solvents (ν_{fs} which is not shown in Table 1 is taken from [2-3]) are acetonitrile, acetone, ethyl acetate, 1,4-dioxane (22250 cm^{-1}), diethyl ether (23520 cm^{-1}), *n*-pentane (25760 cm^{-1}), *n*-hexane and cyclohexane displayed from the right to the left in (a) and from the left to the right in (b). The dielectric constants were taken from [4] and refractive indexes *n* from [5].

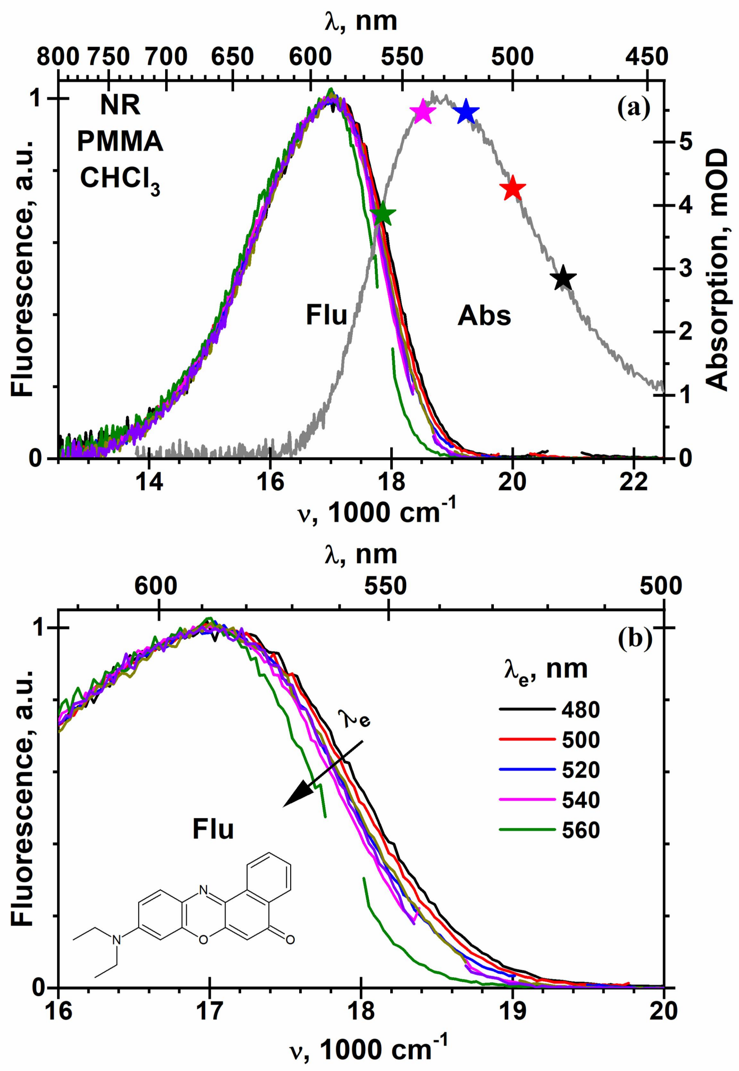

Figure S2: Absorption (a) and fluorescence (a, b) spectra of NR in 480 nm thin film of PMMA (molar mass 350 kg/mol) at different excitation wavelengths (λ_e). The film was prepared by spin coating of PMMA solutions in chloroform ($CHCl_3$) on the 20x20x0.15 mm^3 glass doped with NR. The concentration of NR in the PMMA film of 3.2 mM was calculated from the optical density (panel (a)) and thickness of PMMA film with the molar extinction coefficient NR in 1,4-dioxane of 38000 M^{-1}cm^{-1} [6]. The λ_e values are indicated with stars on the absorption spectrum in panel (a). The regions λ_e ± 5 nm in the fluorescence spectra (a, b) are not shown due to overlap with strong scattering excitation light.

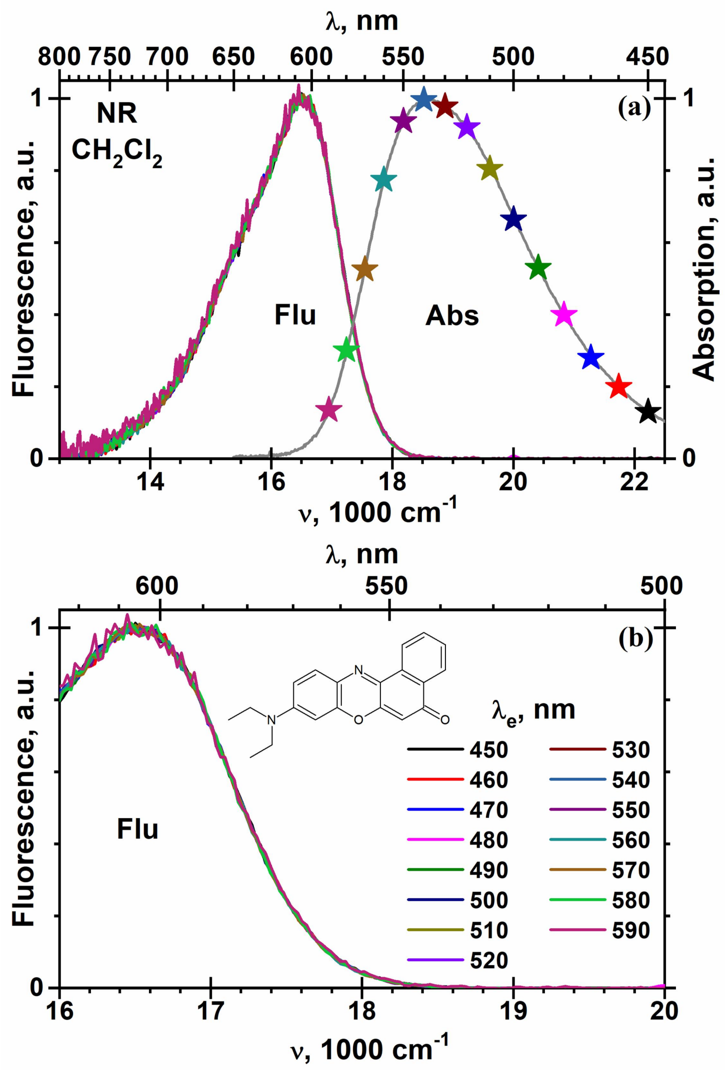

Figure S3: Absorption (a) and fluorescence (a, b) spectra of NR in dichloromethane (CH_2Cl_2) at different excitation wavelength (λ_e) at 25 °C. The λ_e values are indicated with stars on the absorption spectrum in panel (a).

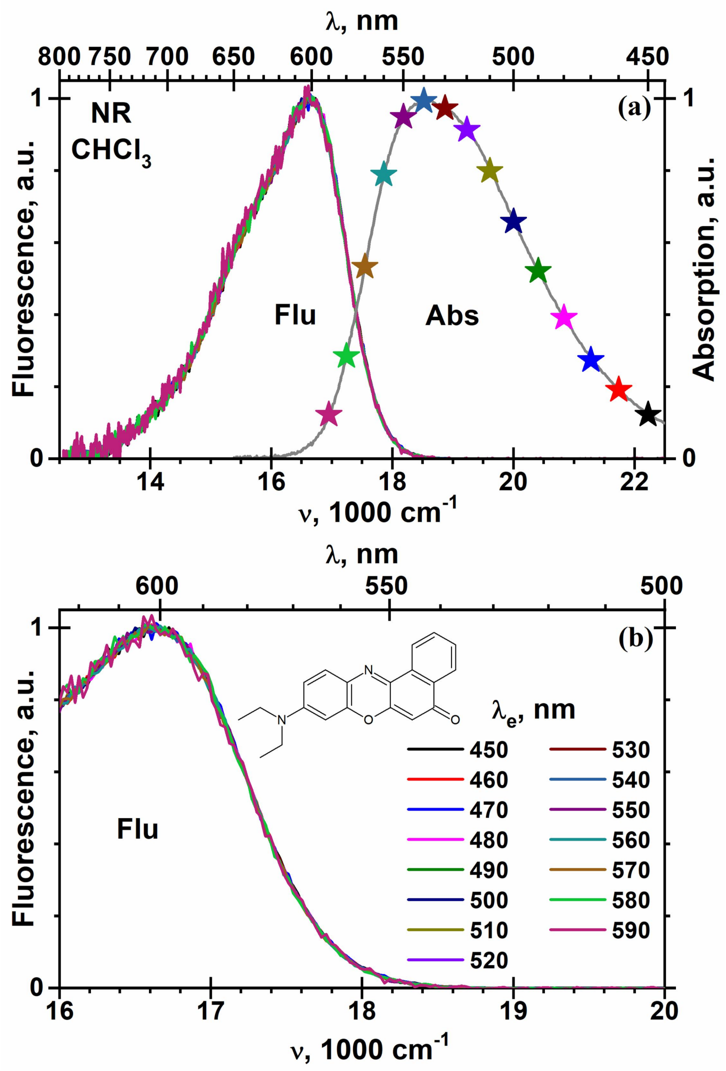

Figure S4: Absorption (a) and fluorescence (a, b) spectra of NR in chloroform ($CHCl_3$) at different excitation wavelengths (λ_e) at 25 °C. The λ_e values are indicated with stars on the absorption spectrum in panel (a).

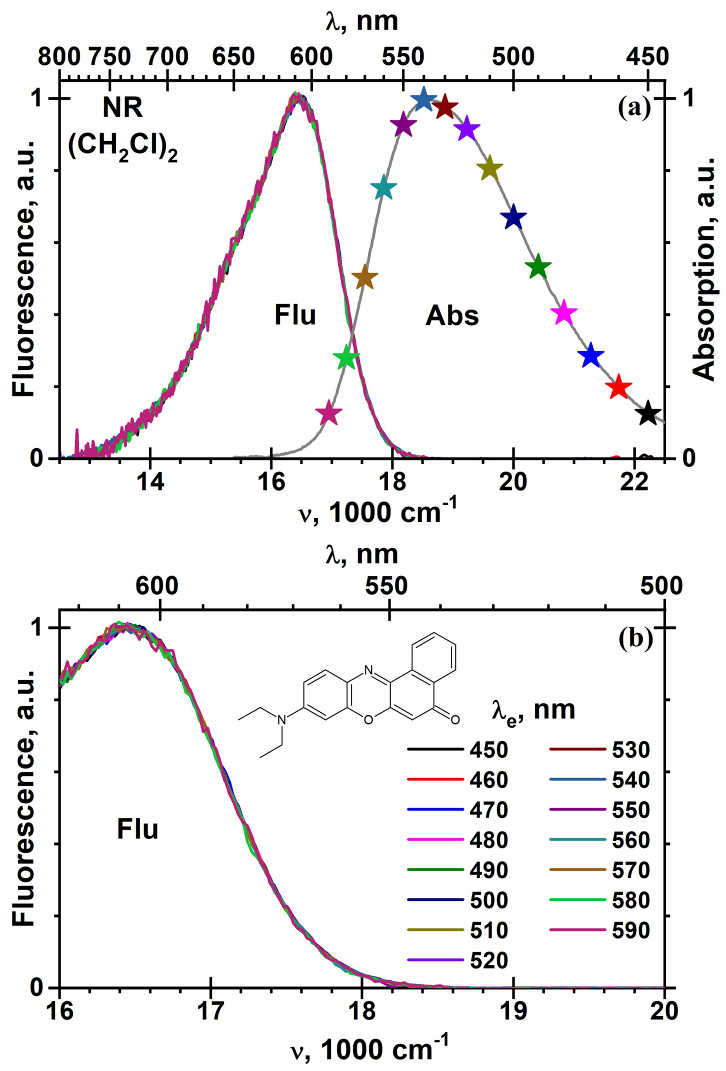

Figure S5: Absorption (a) and fluorescence (a, b) spectra of NR in 1,2-dichloroethane ($(CH_2Cl)_2$) at different excitation wavelengths (λ_e) at 25 °C. The λ_e values are indicated with stars on the absorption spectrum in panel (a).

References

1. Deye, J. F.; Berger, T. A.; Anderson, A. G., *Anal. Chem.* **1990,** *62* (6), 615-622. doi:10.1021/ac00205a015.
2. Zachariasse, K. A.; Druzhinin, S. I.; Bosch, W.; Machinek, R., *J. Am. Chem. Soc.* **2004,** *126* (6), 1705-1715. doi:10.1021/ja037544w.
3. Galievsky, V. A.; Druzhinin, S. I.; Demeter, A.; Jiang, Y.-B.; Kovalenko, S. A.; Lustres, L. P. r.; Venugopal, K.; Ernsting, N. P.; Allonas, X.; Noltemeyer, M.; Machinek, R.; Zachariasse, K. A., *ChemPhysChem* **2005,** *6* (11), 2307-2323. doi:10.1002/cphc.200500267.
4. Static Dielectric Constants of Pure Liquids and Binary Liquid Mixtures. In *Landolt-Börnstein. Numerical Data and Functional Relationships in Science and Technology - Group IV Physical Chemistry*, Madelung, O., Ed. Springer: Berlin, 1991; Vol. 6.
5. Optical Constants. Refractive Indices of Organic Liquids. In *Landolt-Börnstein. Numerical Data and Functional Relationships in Science and Technology - Group III Condensed Matter*, Madelung, O., Ed. Springer: Berlin, 1996; Vol. 38B.
6. Taniguchi, M.; Lindsey, J.S., *Photochem. Photobiol.* **2018**, 94 (2) 290-327. doi:10.1111/php.12860.
